# The Current Testing Protocols for Biomechanical Evaluation of Lumbar Spinal Implants in Laboratory Setting: A Review of the Literature

**DOI:** 10.1155/2015/506181

**Published:** 2015-02-15

**Authors:** Sabrina A. Gonzalez-Blohm, James J. Doulgeris, William E. Lee, Thomas M. Shea, Kamran Aghayev, Frank D. Vrionis

**Affiliations:** ^1^NeuroOncology Department, H. Lee Moffitt Cancer Center & Research Institute, Tampa, FL 33612, USA; ^2^Department of Chemical & Biomedical Engineering, University of South Florida, Tampa, FL 33620, USA; ^3^Departments of Neurosurgery and Orthopedics, Morsani College of Medicine, University of South Florida, Tampa, FL 33613, USA

## Abstract

*In vitro* biomechanical investigations have become a routinely employed technique to explore new lumbar instrumentation. One of the most important advantages of such investigations is the low risk present when compared to clinical trials. However, the best use of any experimental data can be made when standard testing protocols are adopted by investigators, thus allowing comparisons among studies. Experimental variables, such as the length of the specimen, operative level, type of loading (e.g., dynamic versus quasistatic), magnitude, and rate of load applied, are among the most common variables controlled during spinal biomechanical testing. Although important efforts have been made to standardize these protocols, high variability can be found in the current literature. The aim of this investigation was to conduct a systematic review of the literature to identify the current trends in the protocols reported for the evaluation of new lumbar spinal implants under laboratory setting.

## 1. Introduction

For several years, spinal instability has been defined in terms of biomechanical alteration to any spinal element that could affect the vertebral column stability. Even though the previous definition may seem an intuitive concept, quantifying spinal instability and advising the best treatment, conservative or surgical, represents a challenge for the medical and scientific community.

The fact that spinal fusion surgeries have increased by 3.3-fold from 1998 to 2008 [1] motivates the development and evaluation of novel spinal implants. The performance of a spinal implant has been historically addressed by biomechanically comparing, in laboratory settings, the device with an existing one and/or by evaluating the clinical outcomes from randomized clinical trials. On one hand, clinical trials require rigorous regulatory approvals to ensure device's safety (prior to implantation), which could involve significant cost, time, and risk. On the other hand, laboratory testing could provide valuable information that could be further utilized if standard testing protocols are implemented; however, biomechanical testing protocols may vary widely.

Biomechanical investigations typically include analyzing the load-displacement behavior of one or more functional spine unit (FSU) under specific conditions, since a FSU (two adjacent vertebral bodies, intervertebral disc and associated ligaments) is considered the smallest spinal unit representing the mechanical behavior of the entire spine.

An inspection of the literature indicated that the protocols used for biomechanical studies differ with regards to issues such as torque, preload (magnitude and direction), and load rate, parameters that dictate the behavior of the load-displacement curves analyzed. Among the reasons explaining protocols' variability are testing machine restrictions [2], design, and operation variability, which results in findings that may be difficult to compare. Thus, protocol standardization can help reduce result variability between research groups. The objective of this investigation was to systematically review actual testing protocols used in contemporary lumbar spine biomechanical testing.

## 2. Materials and Methods

### 2.1. Electronic Search

A systematic search through PubMed database was conducted on September 2013 using the following terms in the “PubMed Advance Search Builder”:* English* (language),* human* (title/abstract),* lumbar* (title/abstract), and* range of motion* (title/abstract). The term* range of motion* (ROM), specifically, was expected to be mentioned in the vast majority of cadaveric spine biomechanics investigations and was considered to refine the search by helping excluding laboratory investigations on isolated lumbar spinal elements (i.e., vertebra, intervertebral disc, or ligaments). Titles and abstracts meeting the inclusion criteria were selected for further evaluation. Then, the (Materials and/or) Methods' section from the manuscripts of all titles/abstracts selected were tabulated for final evaluation.

### 2.2. Inclusion and Exclusion Criteria

Publications were considered if they were* in vitro* investigations published between September 2008 and September 2013 (5 years). For further consideration, the article had to involve the evaluation of (a) at least one human lumbar or thoracolumbar cadaveric FSU and (b) at least one spinal treatment (injury, surgical procedure, or spinal implant) and (c) its comparison with the “normal” spinal behavior or with other specific condition. Articles were excluded if (a) the FSU(s) within the testing sample involved the cervical, upper thoracic, or middle thoracic region(s), (b) the biomechanical analysis was assessed in isolated FSU elements (i.e., studies on isolated intervertebral discs, ligaments, and anterior column units were excluded), (c) neither a spinal implant nor injury/surgical condition was evaluated (i.e., comparisons of human versus animal biomechanics were excluded), or (d) biomechanical data discussed in the published article was derived from the literature (i.e., articles comparing a finite element model with previously published laboratory data were excluded). Several articles [2–21] retrieved from manual searches were also included in this investigation in order to expand the discussion on relevant topics.

The explanatory variables retrieved from all articles meeting the inclusion criteria are described in Table 1.

## 3. Results

**Figure 1:**
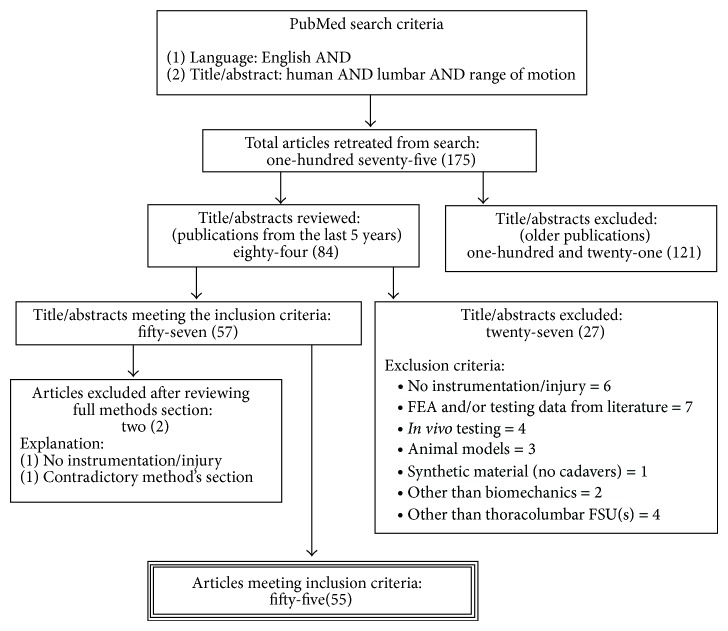
Schematic representation of articles selection process.

Figure 1 illustrates a flowchart of the selection process while Table 2 summarizes the explanatory variables extracted from each investigation. All explanatory variables will be discussed separately for the single- and multisegmental investigations, unless clearly stated. Likewise, percentages presented are with respect to each group specifically, unless otherwise stated.

### 3.1. Single versus Multiple FSUs

Most lumbar* in vitro* biomechanical investigations (83.6%) described the use of multisegmental human spines, with almost 50% including all lumbar vertebrae in their testing segment: L1–L5 [40, 50, 61, 76], L1-pelvis [63, 73], L1-sacrum [35, 39, 41, 44, 45, 51, 57, 64, 65, 75], T12-L5 [32], and T12-Sacrum [37, 62, 72]. All multisegmental investigations used the same spinal levels throughout their samples, except the one performed by Kaibara et al. [52] that included four L2-S1 and three L3-S1 segments. On the other hand, only three (3) [23, 24, 28] of the nine (9) single FSU publications used the same spinal level among their samples.

### 3.2. Operative Level

Most multisegmental studies involved one [32, 33, 35, 36, 38, 40, 42, 44–49, 51–56, 59, 65, 66, 68–70, 72, 74, 75] (60.9%) or two [34, 37, 39, 41, 43, 50, 60, 62, 64, 67, 71, 76] (26.1%) operative level(s) in their testing sample. Moreover, the L3-L4 and the L4-L5 were the most frequent operative levels used in multisegmental investigations (58.7%) that included one or more spinal segment as their operative level(s). On the other hand, the L2-L3 and/or L4-L5 were the most common segments used in single FSU studies (78%).

### 3.3. Loading Protocol: Moment versus Displacement

The* flexibility*,* hybrid*, and* stiffness* protocols were the three protocols used in 80.4%, 17.4%, and 2.2%, respectively, of the multisegmental investigations, while all single FSU studies described a torque-control (*flexibility*) protocol.

### 3.4. Torque and Preload Standards

Preload and torque combinations are summarized in Table 3. A follower compressive preload of 400 N during FE motion was used in thirteen (13) [44–50, 56, 61, 65, 69, 70, 76] of the nineteen (19) multisegmental publications reporting the use of some preload in at least one of their flexion-extension (FE) tests. There were four (4) publications [52, 53, 59, 60] that mentioned the performance of an additional test where a 400 N follower compressive load was applied through a notched belt looped oriented midsagitally over the specimen using a compression-flexion apparatus, which were not included in Table 2 under the* preload* classification.

Among the fifteen (15) multisegmental investigations implementing more than one protocol, the one combining (a) 0 N of preload during FE, lateral bending (LB) and axial rotation (AR) and (b) some follower preload during FE [34, 47–49, 62] was frequently seen. It was also observed that numerous investigations (21 of 46) did not mention a preload paradigm, which was distinguished from those studies explicitly stating that 0 N of preload (14 of 46) was used (Table 2).

In the case of single FSU investigations, only Sangiorgio et al. [27] described the use of more than one preload protocol, which consisted of (a) 0 N of compressive load during FE, LB, and AR and (b) 700 N of follower compressive load during FE.

Two publications described the used of an* off-centered load* protocol: (a) a single FSU study that used a combination of 200 N of axial load with 5.0 Nm of torque [29] and (b) a multisegmental study that used a combination of 55 N and 5.5 Nm (in an additional test) [34].

All investigations concentrated on FE, LB, and AR motions, except for one publication that excluded AR testing [29] and one that included compounded motions (oblique front-right and -left, oblique back-right and -left) [24] as additional tests. Moreover, implementing the same torque/displacement limits for all motions (FE, LB, and AR) was a common practice (89% of all 55 articles), where 7.5 Nm was the most frequent moment applied in both single- (44.4%) and multisegmental studies (47.8%, including two articles that described the use of the* hybrid* protocol [39, 67]), followed by 8.0 Nm (21.7%, including three articles that used the* hybrid* protocols [35, 41, 56] in multisegmental studies). On the other hand, only five (5) multisegmental publications [32, 44, 58, 61, 65] mentioned the application of different load/displacement magnitudes for FE, LB, and AR motions: (a) 8 Nm in flexion, 6 Nm in extension, and LB and 5 Nm in AR [44, 61, 65], (b) 8 Nm in flexion and LB and AR and 6 Nm in extension [58], and (c) 15 deg in FE and 10 deg in LB and AR [32].

Several single- (5) [24, 25, 27, 29, 30] and multisegmental (19) [33, 34, 37, 38, 44, 47–49, 52, 53, 59–62, 64–66, 71, 75] investigations implemented more than one testing protocol. For this reason, publications using two protocols could be listed in Table 2 more than once under the* preload* characteristic. However, only those additional protocols using a small number of cycles (similar to nonfatigue biomechanical testing) were considered in the table and will be discussed in this investigation. On the other hand, additional tests including pure compression [37, 75], fatigue [38], cyclic [30, 64], failure [29, 38], and shear [25] will not be discussed. Thus, this leaves 17 investigations, 2 from single- and 15 from multisegmental, to be considered with more than one testing protocol in the following discussion.

### 3.5. Loading Type and Rate

All single (100%) and several multisegmental (56.5%) studies described the application of the load dynamically, while 21.7% of the multisegmental studies did not specify any protocol. From the twenty-six (26) multisegmental investigations using a* dynamic* protocol, 53.8% reported a rate in degree per second (deg/sec), 30.8% in newton-meter per second (Nm/sec) and 15.4% did not report the rate at which the load was applied. The most common rates were between 1 and 3 deg/sec. Moreover, the* quasistatic* protocol was only used in multisegmental studies (in 21.7% of the 46 articles), where the step size was reported in Nm, commonly as 1.5 Nm, except for one article that did not specify the actual step size (Table 2). In terms of the length of the step, most investigations reported 45 sec [33, 38, 52, 59, 60] to allow creep deformation, while 30 sec [64, 73] and 60 sec [58] were also seen. For single FSU studies, on the other hand, there was no evident trend for reporting the load rate as deg/sec or Nm/sec (Table 2), and the following ranges were seen: 0.1–0.5 Nm/sec and 0.05–1.08 deg/sec, with the exception of the investigation performed by Clair et al. [29] that reported the rate as 10 N/sec (off-centered loading method) (Table 2).

### 3.6. Biomechanical Testing Apparatus

**Figure 2:**
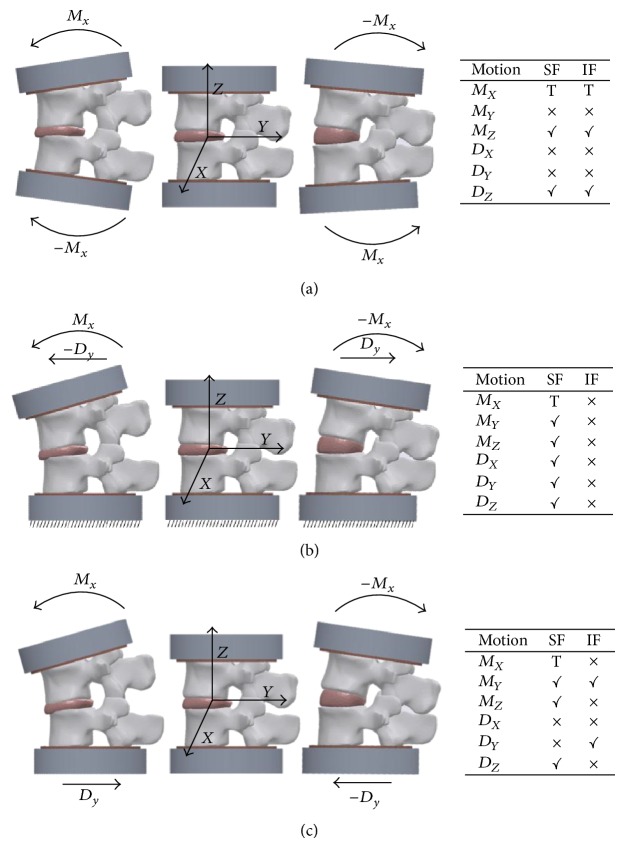
Illustration of three different machines setup during flexion-extension motion: load applied to superior and inferior frames (a), load applied to the superior frame with fixed bottom frame (b), and load applied to superior frame with planar motions in the bottom frame (c). “T” symbol, “letter x” symbol, and “checked” symbol mean torque applied for inducing motion, motion restricted, and motion allowed, respectively. SF = superior frame. IF = inferior frame. *M* = moment. *D* = displacement.

The following testing machines were described: (a) fixed inferior frame and load applied through the superior frame (allowing free motion in all other planes) (~62%) [23–25, 29, 31, 33–36, 39, 40, 44, 47–53, 55, 57, 62–65, 68–74, 76], (b) planar joint on the inferior frame with load applied through superior frame (allowing free motion in all other planes) (~13%) [26, 27, 30, 41–43, 45], and (c) load applied through both superior and inferior frames (constraining motions along all other planes restricted during testing) (~11%) [37, 46, 56, 69, 70, 75] (Figure 2). One article described a compression-bending machine for their FE, LB, and AR tests [29]. Approximately 18% of the articles were mentioned to have previously described the machine or provide minimal information to describe the functionality of the apparatus [22, 28, 32, 38, 54, 58, 59, 61, 66, 67].

### 3.7. Precondition Cycles

The use of 2 cycles for precondition was the most common practice described (Table 2). Only two publications described the implementation of precondition cycles using a different load-protocol than that used for the actual biomechanical test that was analyzed: Laws et al. [58] reported the use of 80% of the torque during the three precondition cycles in a multisegmental study and Sangiorgio et al. [27] described 5 precondition cycles of 350 N of compressive axial load before the flexibility test.

### 3.8. Simulated Injury/Instrumentation

Most biomechanical investigations (94.5%) included the evaluation of at least one spinal implant/instrumentation. Those that did not include any instrumentation were intended to evaluate an injury simulated by the resection of some tissue. It is a common practice to include an injured model in single- (88.9%) and multi- (60.9%) biomechanical investigations; however, only 50% of the articles described the simulation of an injury before instrumentation quantified the injury biomechanically.

### 3.9. Biomechanical Variables

Besides ROM, neutral zone quantification was the second most common parameter assessed biomechanically, followed by intradiscal pressure and the ratio of load to displacement (stiffness). Less than 5% of the investigations included BMD as part of their inclusion criteria for their specimens' selection.

## 4. Discussion

*In vitro* testing has become an important area for investigating the mechanics of the human spine and the performance of new spinal implants and surgical procedures. Developing standard testing protocols is essential for critically comparing findings between studies and for extrapolating their contribution for future discussion. Controversy has always existed about predicting* in vivo* clinical performance from* in vitro* biomechanical results since* in vitro* reproduction of* in vivo* conditions, such as complex load distribution and muscle reactive forces, can be challenging. However, biomechanical investigations involving human cadaveric spines are well-accepted for comparative analysis (e.g., comparing the performance of two or more spinal instrumentation). Moreover, if the results can be interpreted together with clinical data, some assumptions in terms of the instrumentation's clinical performance could be made.

### 4.1. Single versus Multiple FSUs

Animal models have shown how the mechanics of individual FSUs differ from the mechanics of multisegmental specimens [2, 5]; however, this finding should be carefully interpreted. Selecting the number of FSUs depends strictly on the experimental setting; the greater the number of FSUs contained within the testing sample, the larger the number of variables to be accounted. Thus, it should not be necessarily implied that multisegmental models are better just because they emulate “better”* in vivo* scenarios. The loading method utilized, for example, could influence the selection of the specimen length; if an eccentric axial compression load is used, the by-product bending moments will undesirably increase with the specimen length [2]. On the other hand, if a multilevel spinal device is involved in the investigation, a multisegmental specimen is required. Even though there is a great effort on reproducing* in vivo* conditions during* in vitro* testing, the interpretation of* in vitro* results is somehow limited to comparative* in vitro *analysis between two or more conditions. Thus, both single- and multisegmental studies are well-accepted, although it is advisable to (1) provide a rationale for the length of the specimen, (2) maintain the design (specimen's length) throughout testing (when feasible), and (3) disclose any limitation attributed to the specimen's length (if any).

Most multisegmental studies consistently use the same levels in their testing sample while including different spinal levels in single FSU investigations is a common practice. According to a study performed by Posner et al. [16], there was evidence of the L5-S1 segment having different biomechanical performance under axial preload and flexion motion compared to the L1-L2 and the L3-L4 segments, which intuitively suggest that having L5-S1 segments in a single FSU investigation that includes different spinal levels could increase the variability in the measurements for the aforementioned motions. From the single FSU investigations included in this review, only one described the use of L5-S1 segments along with other lumbar segments [30].

### 4.2. Operative Level

Generally, the operative level is selected based on clinical grounds, but specimen's availability is also a factor. The L4-L5 and L3-L4 segments were the most common operative levels seen (Table 2) while the upper lumbar segments, T12-L1 and L1-L2, were the least common.

### 4.3. Loading Protocol: Moment versus Displacement

Three universal protocols are commonly seen in spine biomechanics: the* flexibility*,* stiffness*, and* hybrid* protocols. The* flexibility* protocol, defined by Panjabi et al. in 1976 [12], is the most common used for* in vitro *biomechanical testing, for both single and multisegmental spinal models (Table 2). The reasoning behind this protocol is that by applying a known load and altering the cadaveric segment's condition (by simulating an injury, surgical procedure, or instrumentation), the effects of the treatments can be quantified in terms of displacement's changes with respect to any previous condition.

Conversely, the* stiffness* protocol requires a controlled-displacement to be applied in a certain plane and the load is quantified and compared among treatments [13]. Only one [32] of the 55 investigations included in this review followed this protocol. The rationale behind the* stiffness* protocol is that spinal motion is intuitively thought to be maintained after surgery in order to accomplish usual activities of daily living. Thus, a redistribution of the load along the spine should occur in order to achieve the same goal: preoperative displacement/position. However, the drawback of this protocol includes the possibility of exposing the spinal segment to excessive motion after certain treatments (e.g., rigid instrumentation), which could damage the specimen and limit data reproducibility. Moreover, the validity of this protocol relies on the displacement input to be maintained along the axis of rotation throughout all tests, which is likely to be altered by (1) the natural viscoelastic behavior of the spine (deformation) and (2) instrumentation [11].

The most recent protocol proposed is the* hybrid* [11] and was the second most common used in multisegmental investigations (Table 2). This protocol recommends the use of entire mobile regions of the spine (i.e., T12-Sacrum) and consists of three steps: (1) the application of unconstrained pure loads to the intact (control) spine, (2) application of unconstrained pure loads to the treated spine (by implant/instrumentation) until ROM equals that of the intact testing (from step (1)), and (3) statistical comparison of the biomechanical parameters between conditions to quantify adjacent-level effect [11]. The development of this protocol was justified by the hypothesis that the adaptive response of the spine after spinal instrumentation is to attempt to restore natural motion [11]. The* hybrid* protocol differs from the* stiffness* protocol in that it consists of applying a specific load to the intact condition and then using the “intact” displacement results (baseline) as the input for testing further treatments and evaluating adjacent level displacements, while the* stiffness* protocol maintains the principle of load distribution comparison among treatments by applying the same angular displacement to all conditions.

According to Panjabi [11], the major limitation of the* flexibility* tests on accessing adjacent-level effects is that this protocol uses pure moments, which produces the same moment along the spinal segment. Thus, the response of adjacent levels will not be affected by instrumentation. However, when adjacent-level effect needs to be addressed from a torque-control protocol, normalizing the data (i.e., ROM) with respect to the intact condition and analyzing adjacent-level motion as a percentage of the global motion are a viable option [65]; if the statistical comparisons will be performed on normalized data, the type of tests should be properly selected, especially if comparison of global ROM with respect to the intact condition is needed.

Although the* hybrid* protocol may be seen as the most suitable protocol for multisegmental testing, a new concern arises: the selection of the appropriate moment to be applied to the intact (control) specimen so that the load will not compromise any fundamental structure when testing the following conditions. To illustrate this concern, consider an extreme situation: an L2-L3-L4-L5 pedicle screw system that needs to be evaluated in a T12-sacrum segment. According to the* hybrid* protocol, a moment is initially applied to the intact (noninstrumented) spine and ROM is assessed. Then, instrumentation is implanted and pure unconstrained moments are applied to the instrumented T12-sacrum segment until global (T12-sacrum) motion equals that of the intact condition test. Intuitively, exposing the instrumented condition (long fusion construct) to the ROM achieved during intact testing could compromise the integrity of the spinal segment (especially that of the noninstrumented levels, T12-L2 and L5-S1). One way to overcome this concern would be by carefully selecting the load's magnitude applied at the intact condition, which may be different than that used in* flexibility* protocols. To our knowledge, this has not been addressed. Thus, the* hybrid* protocol should be cautiously considered and further investigation is needed to determine moment limits for the intact segments, probably in terms of the number of levels instrumented. Thus, without moment limit definition for the* hybrid* protocol, using a torque-control protocol may be safer and more conservative for long fusion constructs.

### 4.4. Torque and Preload Standards

#### 4.4.1. Preload

It is well-known that spinal muscles provide an important degree of stability to the spine; however, simulating muscle's response during* in vitro *testing is a great challenge. A common method for minimizing the absence of muscle's forces during* in vitro* testing is by incorporating a compressive preload. The selection of an appropriate compressive preload method and magnitude seems to be more critical in multisegmental spinal models than in single FSU models since its application can derive larger unwanted forces due to a more complex mobile structure (i.e., degree of lordosis or deformation); however, it has been shown how the preload application method and magnitude can also affect artifact reaction moments and shear forces in single FSU testing [3]. Cripton et al. [3] emphasized how by constraining the compressive preload applied, the artifact moments can be reduced at the cost of less moment in AR and greater shear forces in flexion, extension, and LB, for single FSU, during* in vitro *experiments.

Three main paths for the compressive load have been discussed in the literature: vertical, follower, and off-centered [18]. It has been demonstrated how an average of a vertical compressive load of 88 N is enough to cause a buckling effect in a the lumbar spine during* in vitro* experiments [4]. On the other hand, if the load is applied through a follower path, approximately tangent to the natural curvature of the spine, the lumbar spine can withstand up to 1200 N of load [15], a magnitude that is closer to typical compressive loads resisted by the lumbar spine during* in vivo *conditions, such as in standing (~1000 N) and lifting (>1000 N), as cited by Patwardhan et al. [15]; however, optimization of the follower path is critical for minimizing the effects of artifact moments and by-product shear forces [7, 14, 19].

While not being a direct focus of this review, computer simulations have also been used to validate the biomechanical preload hypotheses. A 3D modeling of the lumbosacral spine demonstrated how spinal muscles can generate compressive follower preloads during standing position to withstand* in vivo* stability [8]. Moreover, a lumbar spine finite element model (FEM) developed by Rohlmann et al. [18] showed that realistic ROM results can be obtained for flexion when using either the no-preload, the follower preload, or the eccentric force model and for extension when using the follower preload model; however, the effects of different compressive preload methods and magnitudes, during lateral bending and axial rotation motion, were not investigated. Moreover, Renner et al. [17] validated (through FEM) that a compressive follower load of 800 N can decrease total ROM by 42% and 26% during LB and AR, respectively.

A follower compressive preload of 400 N or larger has shown to significantly affect the ROM of a whole lumbar segment during flexion-extension motion [14]. This protocol of 400 N follower preload during FE motion was the most common preload magnitude and direction used among publications describing the use of some preload for FE. A follower compressive load applied through a notched belt looped oriented midsagitally over the specimen using a compression-flexion apparatus differs from the general concept of follower path (where the load is approximately tangent to the specimen).

Lastly, the* off-centered* protocol, which consists on applying an eccentric compressive load cyclically to create a compression-bending force, was only included in two publications [29, 34].

#### 4.4.2. Torque/Displacement Limits

There is a general consensus on selecting pure bending moments as the loading condition for* in vitro* biomechanical testing. The benefit of applying pure moments in a multisegmental spinal model is the transmission of a uniform load along the segments, which has been shown to reproduce* in vivo *behavior during intact model testing [20]. Several investigations (~75%) explicitly reported the application of pure moments in their methodology section, consisting of 35 multisegmental [31–36, 38, 39, 41–43, 45, 47, 48, 50–53, 55, 57–64, 66–68, 71–75] and 6 single FSU [23–27, 30] articles.

FE, LB, and AR are the common motions evaluated under* in vitro* biomechanical testing. FE could be probably considered the most relevant motion since it is the default directions chosen when additional tests are performed. This could probably be explained by the relevance this motion has for the lumbar spine in clinical scenarios (i.e., activities of daily living).

#### 4.4.3. Preload-Torque Combination

Several (5) multisegmental [47, 51, 66, 67, 74] and one single FSU [23] investigation mentioned to have followed the testing (*flexibility*) protocol proposed by Wilke et al. [21], which consists on applying pure moments of 7.5 Nm at the cranial or caudal end of a nonosteoporotic spinal segment in FE, LB, and AR, without axial preload. There was 1 investigation that used the combination of 7.5 Nm with no axial preload [52] but did not state explicitly to have followed Wilke et al. protocol. All other publications (14) that applied 7.5 Nm of torque in FE, LB and AR deviated from the protocol recommended by Wilke et al. by (a) not mentioning the use of any preload [33, 37–39, 43, 53, 59, 60, 64, 75] (from where 4 included additional FE testing using 400 N of follower preload [33, 53, 59, 60]), (b) including 400 N of follower load in all motions [46, 50, 76], and (c) including 100 N of axial load for all motions [42]. As previously mentioned, 8 Nm was also used in 10 investigations, from where 4 did not mention the use of a compressive load [35, 41, 57, 73], 4 included 400 N of follower load in all direction [45, 56, 69, 70], and 2 used 0 N of compressive load [34, 68].

There is not a widely accepted preload-torque combination. For example, Dreischarf et al. [6] has suggested that a combination of a follower compressive load of 720 N and pure moments of 5.5 Nm applied to the unconstrained cranial (L1) vertebra in AR, specifically, provides the closest results to* in vivo *situation [6]. However, this combination was not seen in any of the investigations here included.

### 4.5. Loading Type and Rate

Little has been said about the most appropriate rate for* in vitro* testing. However, the viscoelastic, rate-dependent behavior of the spine has been widely discussed, justifying the use of quasistatic and dynamic loading. Even though most investigations reported the application of a load dynamically, both load patterns are seen during activities of daily living; dynamic loads represent activities from functional mobility (walking and standing) while quasistatic loads represent stationary activities such as while sitting, holding a weight, or changing posture.

Due to the viscoelastic behavior of the spine, the rate at which the load is applied is an important factor. Although, the most common rates seen during dynamic and quasistatic testing were 1–3 deg/sec and 1.5 Nm/sec, respectively, a rationale for the rate selection was not provided in the methodology of any of the papers reviewed. The selection of the loading type and rate must be based on the objective of the investigation; if creep deformation wants to be assessed then quasistatic loading could be the best option. On the other hand, if damping response and stiffness patterns are of interest, dynamic loading should be chosen.

### 4.6. Biomechanical Testing Apparatus

Torque application is used to simulate the motions of the spine and is often delivered from custom made equipment, which mainly consists of a superior frame and an inferior frame, actuators (or weights), and load cells. Among the different designs described, both fixed- (Figure 2(b)) and planar-inferior machines (Figure 2(c)) allow 6 degrees of freedom (DoF), while the rotational top-bottom setup (Figure 2(a)) loses 2 DoF if planar/shear motions are constrained. A robot arm [62, 72], superior mounted motors connected to a planar joint [39, 51, 71], and pulleys connected to weights [50, 52, 73, 76] have been used to deliver torques for the fixed bottom setups.

In general, the preload is delivered from either an axial arm or a mass pulley system. A mass pulley system is more likely to be used in the free-top and fixed-bottom machines [44, 48, 62, 65] since the top has several DoF and a mass pulley system can be used to deliver a load that is always normal to the specimen. Conversely, planar bottoms may be more useful for a machine that delivers the preload from an axial arm or servohydraulic actuator since it is difficult to set up a planar mechanism to an axial arm. Thus, the load delivery method may be more related to equipment's availability.

Both preload delivery methods represent the major* in vivo* biomechanical factors. The axial arm preload with planar bottom machine intuitively represents the weight of the torso and will transfer some of the preload to a shear force but may not be the best representation of muscle interactions. Conversely, mass pulley systems connected to a free superior frame with a fixed bottom intuitively represent the muscle interactions but may not be the best representation for torso mechanics and shear forces in the lumbar spine. However, the robot arm free top-fixed bottom setup with the inclusion of a mass pulley system could be customized to simulate torso mechanics and muscle interactions; thus, this customization could potentially be the most suitable machine to represent* in vivo* mechanics.

### 4.7. Precondition

Precondition of a spinal segment is a common practice during* in vitro* biomechanical testing since the natural creep experience by the specimen when exposed to either static or dynamic loading [9] could compromise reproducible data. Most investigations agree on the use of two precondition cycles before the data for the analysis is collected.

### 4.8. Injury versus No-Injury Models

The purpose of most spinal implants and instrumentation is to restore stability after an injury, trauma, or surgical procedure. Including an injury condition is a common practice in single- and multisegmental biomechanical investigations (Table 2); however, the injury is not always assessed biomechanically. A common limitation for attempting to quantify an injury is the risk of plastic deformation during the test, which is proportional to the degree of instability triggered by the injury. One way to overcome this problem is to perform the injury model test lastly [45]; however, if the aim of the investigation is to compare the performance of two or more implants with respect to each other, evaluation of an injury may not be necessary.

The injury model, as well as any other simulated surgical procedure, is assumed to be performed by surgeons or qualified scientists using standard techniques. However, in terms of protocol's standardization, it is recommended that these details are mentioned in the manuscript, especially when discussing novel surgical techniques and/or implanting noncommercially available devices.

### 4.9. Biomechanical Variables

**Figure 3:**
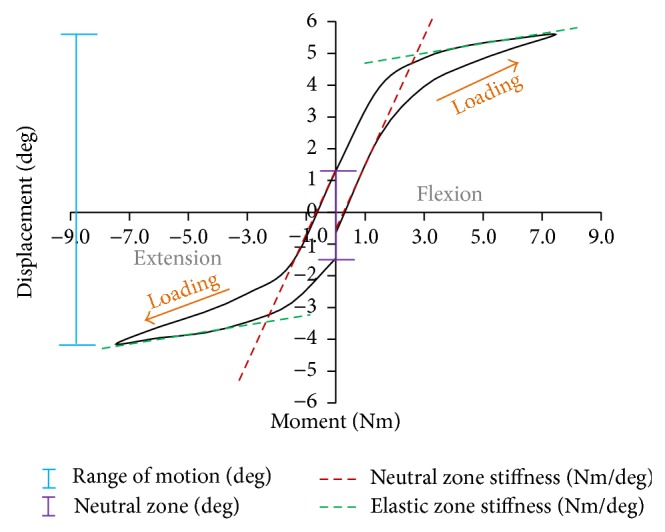
Representation of a load-displacement curve during flexion-extension motion.

Investigating adjacent-level effects has become very popular in spine biomechanics since it is believed that spinal instrumentation, especially fusion devices, can accelerate adjacent level degeneration. Measuring intradiscal pressure and bone strain (i.e., at facet joints or laminae) are some of the parameters used for attempting to predict adjacent degeneration from* in vitro *testing; however, from the literature search performed, ROM (Figure 3) is still the most common parameter (included in all biomechanical investigations) to predict* in vitro *instability. Nevertheless, ROM was included in the search engine which may suggest some bias towards this statement.

The neutral zone (deg) was the second most common parameter evaluated among the articles reviewed in this investigation (Table 1). This concept, introduced by Panjabi in 1992 [10], attempts to quantify the motion around the neutral posture, where ligaments offer little resistance (Figure 3); however, especial care should be taken when trying to quantify this parameter since the measurement's error could be significant for small values, which could occur especially in single FSU studies. In other words, quantification of the neutral zone could require (a) high measurement precision in order to establish accurate statistical comparisons and (b) proper selection of the compressive preload used to avoid misleading results.

The neutral zone changes could be obscured by including an axial preload since the neutral zone parameter is sensitive to the magnitude of the compressive load, as mentioned by Dickey and Kerr [5]. Thus, the use of preload may reduce the difference among conditions; probably more severely than what it would for ROM. However, it does not necessarily mean the nonpreload is a better model since this may be farther from simulating* in vivo* scenario. Both the purpose of the study and the variables to be evaluated will define torque-preload protocols selected.

Stiffness, as a load-displacement ratio, was also a common parameter included. Wilke et al. [21] defined it as the inverse of the slope of the load displacement curve for (a) the neutral region, known as neutral zone stiffness, and for (b) the elastic region, known as elastic zone stiffness (Figure 3). However, other methodologies for estimating this parameter were reported, such as maximal stiffness (maximum moment applied divided by the maximum displacement achieved) [28, 68] and linear stiffness (from linear region of the load-displacement curve) [50, 76], where the latter is ambiguous since there could be more than one linear portion in a load-displacement curve.

Another important parameter, although not measured biomechanically, is the BMD. The purchase of a spinal implant, especially those involving screws, relies on the bone quality. Likewise, specimens with poor bone density are recommended to be exposed to different* in vitro *loads than those that do not have any sign of bone degeneration [21]. This bone BMD criterion was only considered in 55.6% and 30.4% of the single FSU and multisegmental investigations, respectively.

### 4.10. Limitations

It is important to acknowledge the incidence of more than one publication from the same research group which implies a testing apparatus and similar protocols described more than once. However, the intention of this review was to examine what has been recently published in terms of* in vitro* biomechanical testing for the spinal field. Trying to determine which research group was involved in each investigation would have been a challenge since different affiliations were found for several papers and, moreover, this could be potentially considered as a bias factor for the discussion.

This review did not describe in detail how specimens were prepared nor how and who performed simulated surgical procedures since the main focus of the article was on the mechanics of testing. However, we acknowledge that revising these parameters can further complement this review in the search of standardizing protocols for* in vitro* biomechanical testing for spine surgery.

Although the keywords used in the search engine were selected based on what is commonly seen in the field, this could have restricted the search so several publications of interest could have been neglected.

## 5. Conclusion

Most biomechanical investigations are being conducted in multisegmental spines, but single FSU models are also seen in* in vitro* testing. The L3-L4 and L4-L5 were the most commonly used operative levels; however, the selection of the operative level(s), as well as the number of FSUs included in the testing sample, highly depends on the study design. Furthermore, the use of at least six (6) specimens seems to be common practice.

In terms of the testing machine, a fixed inferior frame with loads applied to the unconstrained superior frame was the most common apparatus described. Moreover, the* flexibility* was the main protocol used followed by the* hybrid* protocol. There is no consensus with regards to the protocol (*dynamic* versus* quasistatic*) and magnitude of the load applied; however, both preload and torque/displacement patterns and magnitudes should be specified, even when their magnitude is zero. Before data recording, the use of two precondition cycles seems to be the common practice.

If the purpose of the biomechanical test is to compare two or more spinal instrumentations, biomechanical assessment of an injury condition may not be necessary. ROM is the base biomechanical parameter; however, neutral zone, intradiscal pressure, and regional stiffness are also frequently used in spine biomechanics analysis.

## Figures and Tables

**Table 1:** Description of explanatory variables retrieved from all articles meeting the inclusion criteria.

**Variable**	**Description**
Number of FSUs	Number of FSUs within testing sample

Operative level	FSU selected for surgical intervention. An article could be classified under more than one operative level category if (a) more than one FSU was involved in the surgical procedure or (b) different operative levels were used along the study.

Number of specimens	Sample size of the investigation, per testing group (if more than one group was considered).

Loading protocol	Protocol selected for biomechanical testing (i.e., load control and displacement control).

Preload	Axial load applied during biomechanical testing of FE, LB, or AR. An article could be classified under more than one preload condition if at least two different protocols were implemented for FE, LB, and/or AR.

Load	Magnitude of the force applied to induce FE, LB, and/or AR. An article could be classified under more than one load if different magnitudes were reported for tested motions

Load type and rate	Load's pattern (continuous versus stepwise) and its velocity during biomechanical testing. An article could be classified under more than one load rate if different velocities were described for two or more motions

Biomechanical testing apparatus	Mechanical properties of testing machine used for biomechanical testing

Precondition cycles	Number of cycles performed before data collection for the analysis

Simulated Injury and/or Instrumentation	Specifies if the article included an “injury” condition and/or instrumentation. Those articles that did not include the evaluation of any instrumentation did mandatorily include the biomechanical assessment of an injury (inclusion criterion).

Common Variables Measured	Parameters evaluated during biomechanical testing. Bone mineral density (BMD) was included in this category although it was not derived from biomechanical testing.

FSU: Functional Spinal Unit; FE: Flexion-Extension; LB: Lateral Bending; AR: Axial Rotation.

**Table 2:** Summary of explanatory variables examined for all *in vitro* biomechanical investigations.

	Single FSU Studies		Multisegmental Studies	
	Number of articles	References	Number of articles	References
Number of FSUs				
Single (1)	**9**	[22–30]	—	
Two or more	—		**46**	[31–76]
Two (2)	—		**5**	[38, 42, 43, 60, 66]
Three (3)	—		**13**	[33, 34, 36, 47–49, 52–55, 67, 68, 74]
Four (4)	—		**12**	[31, 40, 46, 50, 52, 56, 59, 61, 69–71, 76]
Five (5)	—		**13**	[32, 35, 39, 41, 44, 45, 51, 57, 63–65, 73, 75]
Six (6)	—		**13**	[37, 62, 72]

Operative Level^*^				
T12-L1	**2**	[26, 30]	—	
L1-L2	**3**	[26, 29, 30]	**1**	[59]
L2-L3	**7**	[22, 23, 25–27, 29, 30]	**6**	[50, 57, 58, 61, 73, 76]
L3-L4	**4**	[24, 26, 29, 30]	**27**	[31, 32, 34, 40, 41, 45, 47–51, 54, 57, 58, 61–65, 67–69, 72–76]
L4-L5	**7**	[22, 25–30]	**27**	[31, 33–37, 39, 41, 43, 44, 46, 52, 53, 55–58, 60–64, 66, 67, 70, 71, 73]
L5-Sacrum	**1**	[30]	**9**	[31, 37–39, 42, 43, 60, 71, 73]

Number of specimens				
Less than six (6)	**1**	[24]	**4**	[34, 46, 54, 69]
Six (6) or seven (7)	**2**	[22, 23]	**29**	[31–33, 36–39, 42–45, 52, 53, 55, 57, 59–63, 66–68, 70–75]
More than seven (7)	**6**	[25–30]	**13**	[35, 40, 41, 47–51, 56, 58, 64, 65, 76]

Loading protocol^*^				
Flexibility	**9**	[22–30]	**37**	[31, 33, 34, 36–38, 40, 42–54, 57–61, 63–66, 68–70, 72–76]
Hybrid	—		**8**	[35, 39, 41, 55, 56, 62, 67, 70]
Other	—		**2**	[32, 60]

Preload^*^				
None (0 N)	**2**	[23, 27]	**14**	[31, 34, 40, 47–49, 51, 52, 62, 66–68, 71, 74]
Axial	**1**	[22]	**2**	[42, 66]
Follower	—		**8**	[45, 46, 50, 56, 69–71, 76]
Less than 400 N	—		**1**	[71]
400 N	—		**7**	[45, 46, 50, 56, 69, 70, 76]
More than 400 N	—		—	
Follower FE only	**1**	[27]	**10**	[44, 47–49, 53, 59–62, 65]
Less than 400 N	—		—	
400 N	—		**6**	[44, 47–49, 61, 65]
More than 400 N	**1**	[27]	**1**	[62]
Bending-compression	**1**	[29]	**2**	[34, 52]
Unspecified^**^	**5**	[24–26, 28, 30]	**21**	[32, 33, 35–39, 41, 43, 53–55, 57–60, 63, 64, 72, 73, 75]

Torque applied^*^				
Less than 7.5 Nm	**4**	[24–26, 29]	**10**	[31, 40, 44, 54, 58, 61–63, 65, 72]
7.5 Nm	**4**	[22, 23, 28, 30]	**22**	[33, 37–39, 42, 43, 46–53, 59, 60, 64, 66, 67, 74–76]
More than 7.5 Nm	**1**	[27]	**17**	[34–36, 41, 44, 45, 55–58, 61, 65, 68–71, 73]
Displacement control	—		**1**	[32]

Loading type and load rate				
Dynamic loading^*^	**9**	[22–30]	**26**	[31, 32, 37, 39, 41–46, 48, 51, 55–57, 61–63, 65, 66, 68–71, 74, 75]
Less than 1 deg/sec	**2**	[23, 24]	**2**	[32, 45]
1–3 deg/sec	**3**	[23, 25, 28]	**12**	[31, 32, 39, 41, 55, 57, 61, 63, 66, 68, 71, 74]
4 deg/sec	—		**1**	[51]
Less than 0.1 Nm/sec	—		**2**	[37, 75]
0.1–0.5 Nm/sec	**3**	[22, 26, 27, 30]	**6**	[42, 43, 46, 56, 69, 70]
Other	**1**	[29]	—	
Unspecified	—		**4**	[44, 48, 62, 65]
Quasistatic loading	—		**10**	[33, 34, 36, 38, 52, 58–60, 64, 73]
1.0 Nm/sec	—		**1**	[36]
1.5 Nm/sec	—		**6**	[33, 38, 52, 59, 60, 64]
2.0 Nm/sec	—		**2**	[58, 73]
Unspecified	—		**1**	[34]
Unspecified^***^	—		**10**	[35, 40, 48–50, 53, 54, 67, 72, 76]
Precondition Cycles				
One (1)	—		**3**	[41, 47, 48]
Two (2)	**7**	[22–26, 29, 30]	**23**	[31, 32, 37, 39, 40, 42, 43, 46, 55–57, 62, 63, 66–75]
Three (3) or more	**2**	[27, 28]	**9**	[33, 34, 36, 38, 52, 58–60, 64]
Unspecified	—		**8**	[35, 45, 49, 50, 53, 54, 61, 76]
Until 2 Reproducible cycles	—		**3**	[44, 51, 65]
Simulated Injury/Instrumentation				
No Instrumentation	**1**	[25]	**2**	[49, 61]
Instrumentation	**8**	[22–24, 26–30]	**44**	[31–48, 50–60, 62–76]
Injury Described	**8**	[22–24, 26–30]	**28**	[31, 33–41, 43–46, 48, 54–58, 60, 65–67, 69, 70, 74, 75]
*Injury Tested *	**3**	[23, 24, 27]	**15**	[33–37, 39, 40, 44, 45, 54, 55, 57, 66, 67, 69]
Common Variables Measured				
Range of Motion	**9**	[22–30]	**46**	[31–76]
Neutral Zone	**5**	[22, 23, 25, 26, 29]	**10**	[33, 38, 45, 58–60, 65–67, 73]
Elastic Zone	—		**4**	[33, 38, 59, 60]
Intradiscal Pressure	—		**9**	[36, 39, 41, 46, 50, 55, 66, 69, 70]
Stiffness	**3**	[22, 25, 28]	**5**	[50, 58, 65, 68, 76]
Neutral Zone Stiffness	**1**	[22]	**1**	[65]
Elastic Zone Stiffness	**1**	[22]	**1**	[58]
“Other” Stiffness	**2**	[25, 28]	**3**	[50, 68, 76]
Radiological Measurements^****^	**2**	[22, 30]	**5**	[44, 52, 53, 59, 74]
Bone Strain	—		**4**	[32, 46, 60, 70]
Instantaneous Axis of Rotation (IAR)	—		**4**	[33, 36, 59, 60]
Bone Mineral Density	**5**	[22, 26, 28–30]	**14**	[31, 38, 42, 43, 45, 46, 53, 56, 58–60, 64, 67, 73]

^*^Some articles appear more than once under this classification due to the use of more than one testing protocol.

^**^The application of an axial preload was not mentioned hence articles in this category were distinguished from those that stated explicitly the use of 0 N of axial preload.

^***^Includes articles that mentioned to have followed a previously described protocol without providing the information in the methodology of the actual article.

^****^Articles describing manual measurements (i.e caliper measurements) were excluded from this category.

**Table:** (a) Single functional spinal unit

Spinal segment	Number of articles	Torque [Nm] used at indicated preload							
		No preload mentioned	0 [N]	1–100 [N]	200 [N]	300 [N]	400 [N]	600 [N]	700 [N]
T12-L1	2^*^	6.0^2^/7.5							
L1-L2	3^*^	6.0^2^/7.5			5.0 (e)				
L2-L3	7^*^	5.0/6.0^2^ 7.5	7.5/8.0	7.5	5.0 (e)				8
L3-L4	4^*^	3.0/6.0^2^ 7.5			5.0 (e)				
L4-L5	7^*^	5.0/6.0^2^ 7.5^2^	8.0	7.5	5.0 (e)				8
L5-sacrum	1^*^	6.0/7.5							

**Table:** (b) Multiple functional spinal units

Spinal segment	Number of articles	Torque [Nm] used at indicated preload							
		No preload mentioned	0 [N]	1–100 [N]	200 [N]	300 [N]	400 [N]	600 [N]	700 [N]
T12-L2	1^*^	7.5					7.5		
T12-L5	1	(unspecified)							
T12-sacrum	3^*^	5.0/7.5	H					H	
L1–L5	4^*^		3.5/5.0 6.0/8.0				5.0/6.0 7.5/8.0		
L1-sacrum	11^*^	2.5/5.0 6.0/7.5^3^ (H^2^) 8.0^4^ (H^2^)	5.0^2^/6.0^2^ 7.5/8.0^2^				2.5/5.0^2^ 6.0^2^/7.5 8.0^3^		
L1-Pelvis	2	6.0/8.0							
L2–L5	7^*^	5.0	7.5^5^ (H)/8.0				7.5		
L2-S	8^*^	7.5	6.0/7.5 10.0 (H)			10.0 (H)	7.5^2^, 8.0^3^ (H)		
L3–L5	1^*^		7.5				7.5		
L3-S	5^*^	7.5^2^/10.0^2^ (H)	8.0 (e)	8.0 (e)					
L4-sacrum	3	7.5^2^		7.5					

^*^One or more article described two or more testing protocols. This creates a discrepancy between “number of articles” and the number of preload-torque combinations described (i.e., the single-segment article that used L5-sacrum as their testing sample described two different protocols: (A) unspecified preload + 6.0 Nm of torque and (B) unspecified preload + 7.5 Nm of torque).

Superscripts refer to the number of articles describing the indicated load-torque combination.

e: eccentric loading; H: hybrid loading. (i.e., 8.0^4^ (H^2^) = four articles described the used of 8.0 Nm, two of which used Hybrid loading).
